# The first case report of chylous ascites in a twin pregnancy following *in vitro* fertilization: a case report of chylous ascites in twin pregnancy

**DOI:** 10.1097/MS9.0000000000003686

**Published:** 2025-08-08

**Authors:** Nguyen Xuan Hoi, Nguyen Thi Huyen Anh, Phung Thi Ly, Nguyen Dang Tuan

**Affiliations:** a National Hospital of Obstetrics and Gynecology, Hanoi, Vietnam; b Hanoi Medical University, Hanoi, Vietnam; c Vinmec Times City International Hospital, Hanoi, Vietnam

**Keywords:** case report, chylous ascites, pregnancy

## Abstract

**Introduction and importance:**

Chylous ascites is an exceptionally rare condition during pregnancy, typically presenting with nonspecific symptoms. To date, only a limited number of cases have been reported in the literature, and the majority of which occurred in singleton pregnancies.

**Case Presentation:**

We report the case of a 25-year-old woman with a twin pregnancy who was admitted at 32 weeks of gestation with acute abdominal pain and nausea. Due to persistent severe pain, unexplained intraperitoneal fluid, and progressing labor, an emergent cesarean section was performed. Intraoperatively, 2000 mL of milky white fluid was discovered in the peritoneal cavity. Biochemical analysis with markedly elevated triglycerides confirmed the diagnosis of chylous ascites. Postoperative management included broad-spectrum intravenous antibiotics, octreotide injection, and a low-fat diet supplemented with medium-chain triglyceride oil. By postoperative day 7, ultrasound showed complete resolution of ascites, and the patient was discharged in stable condition. No recurrence was noted during 3 years of follow-up.

**Clinical discussion:**

This report presents a rare case of spontaneous chylous ascites in a previously healthy pregnant woman with a twin gestation. Potential mechanism for chylous ascites in our case can be due to enlarged gravid uterus of twin gestation during late pregnancy, which suppress the abdominal thoracic duct of mother and increase the pressure of the duct. Another possible mechanism involves the physiological effects of progesterone during pregnancy. Progesterone is known to induce smooth muscle relaxation, which can lead to dilation of lymphatic vessels and increased lymphatic flow. This vasodilatory effect may predispose to lymphatic leakage and contribute to the development of chylous ascites. The presence of milky white peritoneal fluid combined with a markedly elevated triglyceride concentration (≥ 110 mg/dL, 1945 mg/dL in our case) was key to confirm the diagnosis of chylous ascites. Treatment strategies typically depends on the underlying etiology and may include dietary modification (low-fat, MCT-enriched diet), pharmacologic therapy (somatostatin or octreotide), and surgical interventions in refractory cases.

**Conclusion:**

Chylous ascites should be included in the differential diagnosis of unexplained ascites in twin gestation, particularly when the fluid exhibits a milky appearance. Although chylous ascites resolves spontaneously in the postpartum period, this condition may necessitate the termination of pregnancy and requires a multidisciplinary approach, involving obstetricians, gastrointestinal surgeons, radiologists, and nutritionists, for timely diagnosis and effective management.

## Introduction

Chylous ascites is a rare form of ascites characterized by the leakage and accumulation of lipid-rich lymph into the peritoneal cavity. Although this condition has been described in association with various underlying etiologies such as malignancy, trauma, or infectious processes, it remains exceedingly rare in the context of pregnancy, with only a limited number of cases reported in the literature[1]. The pathophysiological mechanisms responsible for the development of chylous ascites during gestation are not yet fully elucidated. Some cases have been identified as complications arising from pancreatitis^[2,3]^, while others were discovered incidentally during cesarean sections[4]. This article aims to present the first case of chylous ascites occurring during twin pregnancy following *in vitro* fertilization and to provide a review of the current literature regarding its etiology, diagnosis, and clinical management. This case report has been reported in line with the SCARE 2025 criteria[5].

**HIGHLIGHTS**

- Chylous ascites is an exceptionally rare condition during pregnancy, with only a limited number of cases reported in the literature. This article aims to present the first case of chylous ascites occurring in twin pregnancy following *in vitro* fertilization.
- Potential mechanisms for chylous ascites in twin pregnancies can be due to enlarged gravid uterus and physiological effects of progesterone during pregnancy.
- Chylous ascites should be included in the differential diagnosis of unexplained ascites in twin gestation, particularly when the fluid exhibits a milky appearance.
- While chylous ascites is often a benign condition that resolves spontaneously in the postpartum period, it can necessitate the termination of pregnancy.
- Management requires a multidisciplinary approach, involving obstetricians, gastrointestinal surgeons, radiologists, and nutritionists, to ensure timely diagnosis and appropriate treatment.

## Presentation of case

A 25-year-old G0P0 woman with a twin pregnancy, conceived via *in vitro* fertilization, was admitted to our hospital at 32 weeks of gestation with complaints of acute abdominal pain and nausea. Her antenatal course had been unremarkable, with routine prenatal visits and no reported complications. She had no notable past medical or surgical history, and her family history was noncontributory. On admission, physical examination revealed a diffusely distended and tender abdomen with signs of peritonitis. Her vital signs were stable: blood pressure 115/67 mmHg, pulse 87 beats per minute, temperature 37.0°C, and respiratory rate 18 breaths per minute. Fetal heart rate tracing was reactive for both fetuses. Uterine contractions were noted to occur every 2 to 3 min, each lasting approximately 60 s. Pelvic examination indicated that her cervix was 2 cm dilated and completely effaced. Laboratory investigations showed leukocytosis with a white blood cell count of 24.1 × 10^9^/L and an absolute neutrophil count of 20.2 × 10^9^/L. Procalcitonin was mildly elevated at 0.158 ng/mL. Other parameters, including complete blood count, serum biochemistry, coagulation profile, and urinalysis, were within normal limits. Abdominal ultrasonography detected an abnormal amount of free fluid within the peritoneal cavity, the etiology of which was unclear. Given the persistence of severe abdominal pain, the presence of unexplained intraperitoneal fluid, and the progression of labor, an emergent cesarean section was performed.

**Figure 1.**
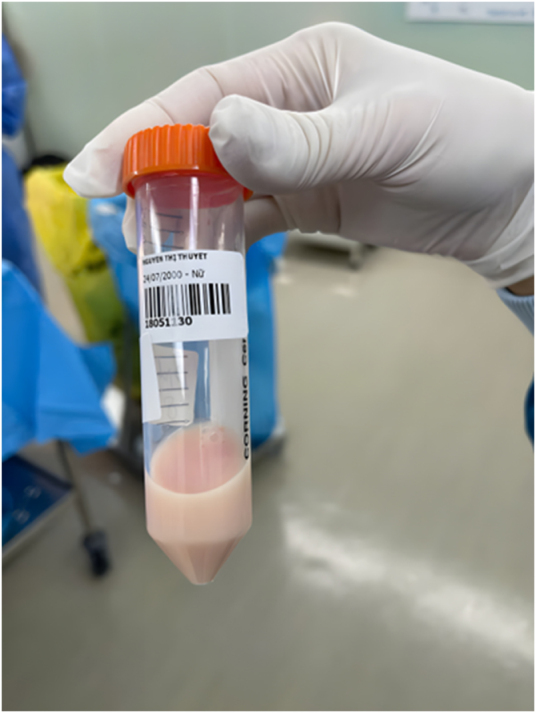
Milky fluid was evacuated from the peritoneal cavity.

**Figure 2.**
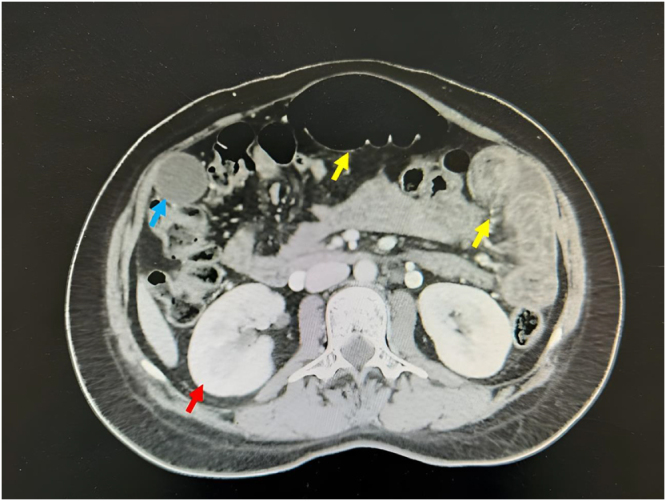
CT scans of the abdomen and pelvis excluded the presence of malignancy or other intra-abdominal pathology (red arrow: kidney, blue arrow: gallbladder, yellow arrows: colon).

Upon entry into the peritoneal cavity, approximately 2000 mL of milky white fluid was observed and subsequently collected for microbiological and biochemical analysis (Fig. 2). Two live neonates weighing 2240 g and 1840 were born with Apgar scores of 7 and 8 at the first and fifth minutes, respectively. The amniotic fluid was clear, and there was no evidence of placental abruption. An intraoperative consultation with a general surgeon was conducted to assess the abdominal organs. No abnormalities were observed during the intervention: the uterus, adnexa, appendix, and stomach appeared normal, the loops of small intestine showed good coloration, preserved peristalsis, and moderate dilatation. Following evaluation, a drainage tube was placed in the peritoneal cavity prior to closure. The drain remained *in situ* until the output decreased significantly and was removed on the day of discharge. Biochemical analysis of milky fluid revealed a markedly elevated triglyceride level (1945 mg/dL) and a mildly elevated lipase level (37.5 U/L), with normal protein and glucose concentrations. Aerobic bacterial cultures were negative. The characteristic turbid, milky appearance of the fluid, in conjunction with the elevated triglyceride level, confirmed the diagnosis of chylous ascites (Fig. 1).

**Figure 3.**
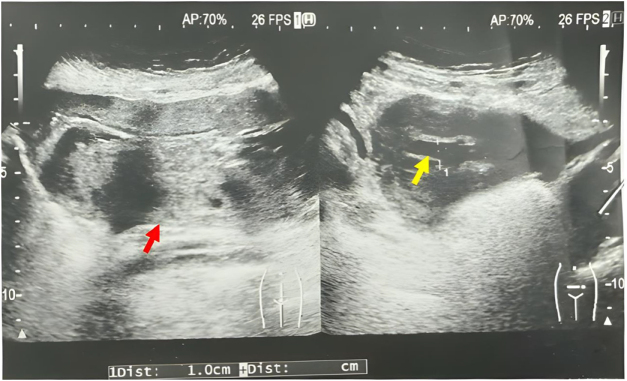
Transabdominal ultrasound demonstrated a normal-appearing uterus (red arrow) with a small amount of fluid in the endometrial cavity (yellow arrow). No free intraperitoneal fluid was detected.

Postoperatively, the patient was administered broad-spectrum intravenous antibiotics, including cefoxitin 1 g and metronidazole 500 mg, and octreotide injection. A low-fat diet supplemented with medium-chain triglyceride (MCT) oil was also initiated to reduce lymphatic flow and facilitate resolution of the chylous ascites. On postoperative day 3, computed tomography (CT) scans of the chest, abdomen, and pelvis were performed to exclude the presence of malignancy, lymphatic malformations, or other intra-abdominal pathology (Fig. 2). Imaging findings were unremarkable, with no evidence of tumor or structural abnormalities. The patient’s postoperative course was uneventful, with gradual resolution of abdominal symptoms. By postoperative day 7, an abdominal ultrasound confirmed the absence of free intraperitoneal fluid (Fig. 3), and the patient was discharged in stable condition. Follow-up over a 3-year period revealed no recurrence of ascites or other complications.

## Discussion

Chylous ascites is a rare clinical entity, and its exact etiology remains incompletely understood. Several mechanisms have been proposed to explain the pathophysiology of chylous ascites:^[2,6–9]^

- **Lymphatic obstruction or disruption**: Obstruction of lymphatic flow due to malignancies (e.g., lymphoma) or trauma to the lymphatic vessels can lead to extravasation of chyle into the peritoneal cavity.
- **Congenital lymphatic malformations**: Developmental abnormalities of the lymphatic system, such as lymphangiectasia or lymphangiomatosis, may cause chyle leakage, particularly in younger patients.
- **Infectious or inflammatory processes**: Conditions such as tuberculosis or pancreatitis may induce lymphatic inflammation or rupture, resulting in chylous effusion.
- **Elevated intra-abdominal pressure during pregnancy**: The gravid uterus may exert mechanical pressure on the cisterna chyli or thoracic duct, especially in multiple gestations, leading to transient lymphatic rupture in susceptible individuals.

Chylous ascites during pregnancy is exceedingly uncommon, with only a limited number of cases documented in the literature. Previous reports have described varying etiologies and clinical presentations of chylous ascites in pregnancy. In cases reported by Zhang et al (2018) and Epelde (2024), chylous ascites was discovered incidentally during cesarean section in a woman with no predisposing risk factors^[4,7]^. In contrast, Yang et al. (2017) and Apikotoa et al (2021) described pregnant women presenting with chylous ascites secondary to acute pancreatitis during the third trimester^[2,3]^. Other proposed mechanisms in pregnancy include mechanical lymphatic obstruction caused by the enlarging uterus, trauma to the lymphatic channels, or the exacerbation of previously unrecognized congenital lymphatic anomalies due to physiological changes in pregnancy. Habek et al. (2005) reported chylous ascites in pregnant patients secondary to pulmonary tuberculosis in childhood with congenital lymphangiectasis[9]. Sun et al (2007) reported chylous ascites identified during cesarean delivery, which was later attributed to a mesenteric tumor diagnosed by postoperative spiral CT imaging[10]. Babic et al (2012) noted spontaneous ascites in a female with morbid obesity at time of cesarean section[11].

This report presents a rare case of spontaneous chylous ascites in a previously healthy pregnant woman with a twin gestation. Potential mechanism for chylous ascites in our case can be due to enlarged gravid uterus of twin gestation during late pregnancy, which suppress the abdominal thoracic duct of mother and increase the pressure of the duct. Such compression can lead to either spontaneous rupture of the dilated engorged lymph vessels or appearance of giant retroperitoneal lymphatic vessel wall, resulting in spontaneous exudation of chyle in the walls of bowel or mesentery under the increased pressure during labor. Another possible mechanism involves the physiological effects of progesterone during pregnancy. In our case, the patient had been receiving 400 mg of progesterone daily for the prevention of preterm labor. Progesterone is known to induce smooth muscle relaxation, which can lead to dilation of lymphatic vessels and increased lymphatic flow. This vasodilatory effect may predispose to lymphatic leakage and contribute to the development of chylous ascites.

In pregnancy, chylous ascites is an extremely rare finding associated with nonspecific symptoms. The most frequently reported symptom is abdominal distension, observed in approximately 81% of cases, followed by abdominal pain or signs of peritonitis in about 11%[12]. Apikotoa et al (2021) reported a case in which the predominant symptom was diffuse abdominal pain accompanied by leukocytosis during pregnancy, a presentation that closely resembles our patient’s clinical course[3]. Additionally, two cases have been reported in which patients developed acute abdominal pain and distension in the immediate postpartum period following an uncomplicated vaginal delivery; exploratory laparotomy in both instances revealed milky peritoneal fluid consistent with chylous ascites^[4,7]^. In the present case, abdominal ultrasonography identified the presence of intraperitoneal free fluid, yet its nature was only clarified intraoperatively. The presence of milky white peritoneal fluid combined with a markedly elevated triglyceride concentration (≥ 110 mg/dL, 1945 mg/dL in our case) was key to confirm the diagnosis of chylous ascites. This emphasizes the importance of intraoperative assessment and biochemical analysis of ascitic fluid when the etiology remains unclear preoperatively.

The current literature provides limited guidance on the optimal management of chylous ascites during pregnancy. Treatment strategies typically depends on the underlying etiology and may include dietary modification (low-fat, MCT-enriched diet), pharmacologic therapy (somatostatin or octreotide), and surgical interventions in refractory cases. Among these, dietary modification remains the first-line approach due to its noninvasive nature and safety profile in pregnancy. A low-fat diet enriched with MCTs is commonly recommended, as MCTs are absorbed directly into the portal circulation, bypassing the lymphatic system and thereby reducing lymph flow. In contrast, long-chain triglycerides, which constitute approximately 95% of dietary fat, are transported via intestinal lymphatics and can exacerbate chyle leakage. If dietary measures alone are insufficient, pharmacologic therapy with somatostatin or its synthetic analogue, octreotide, may be considered. These agents have been shown in several case reports to reduce lymph production and facilitate resolution of chylous ascites. In non-pregnant patients or severe refractory cases, interventional procedures such as lymphatic embolization, surgical ligation, or placement of a transjugular intrahepatic portosystemic shunt may be warranted, particularly when cirrhosis or malignancy is implicated[12]. However, the safety and applicability of these invasive interventions during pregnancy require careful multidisciplinary consideration.

In this case, MCT-enriched diet combined with octreotide injection were implemented immediately postoperatively, which led to significant clinical improvement and resolution of symptoms. Although pharmacologic therapies such as somatostatin or octreotide have shown promise in the management of chylous ascites, there is limited evidence regarding their safety and efficacy during pregnancy. Consequently, therapeutic decisions should be carefully individualized, balancing maternal benefit against potential fetal risks. A review of documented cases suggests that termination of pregnancy often leads to complete resolution of chylous ascites. This observation raises the possibility that the gravid uterus may contribute to lymphatic obstruction or rupture, and that decompression following delivery facilitates recovery. However, the role of pregnancy termination as a definitive treatment for chylous ascites remains unclear, and further studies are needed to establish evidence-based guidelines for managing this rare condition in pregnant women.

## Conclusions

Chylous ascites is an exceedingly rare and challenging condition to diagnose during pregnancy. It should be included in the differential diagnosis of unexplained ascites in twin gestation, particularly when the fluid exhibits a milky appearance. While chylous ascites is often a benign condition that resolves spontaneously in the postpartum period, it can necessitate the termination of pregnancy. Management requires a multidisciplinary approach, involving obstetricians, gastrointestinal surgeons, radiologists, and nutritionists, to ensure timely diagnosis and appropriate treatment.

## Data Availability

Not applicable.
